# Activated fibroblasts enhance cancer cell migration by microvesicles-mediated transfer of Galectin-1

**DOI:** 10.1007/s12079-021-00624-4

**Published:** 2021-05-22

**Authors:** Alessandra Toti, Alice Santi, Elisa Pardella, Ilaria Nesi, Richard Tomasini, Tommaso Mello, Paolo Paoli, Anna Caselli, Paolo Cirri

**Affiliations:** 1 Dipartimento di Scienze Biomediche Sperimentali e Cliniche “Mario Serio”, Università degli Studi di Firenze, Viale Morgagni 50, 50134 Firenze, Italy; 2 Cancer Research UK Beatson Institute, Glasgow, UK; 3 INSERM, U1068, Centre de Recherche en Cancérologie de Marseille, Institut Paoli-Calmettes, CNRS, UMR7258, Université Aix-Marseille, Marseille, France

**Keywords:** Cancer associated fibroblasts, Galectin-1, Microvesicles, Extracellular vesicles, Cell migration

## Abstract

Cancer-associated fibroblasts (CAFs) are one of the main components of the stromal compartment in the tumor microenvironment (TME) and the crosstalk between CAFs and cancer cells is essential for tumor progression and aggressiveness. Cancer cells mediate an activation process, converting normal fibroblasts into CAFs, that are characterized by modified expression of many proteins and increased production and release of microvesicles (MVs), extracellular vesicles generated by outwards budding from the cell membrane. Recent evidence underlined that the uptake of CAF-derived MVs changes the overall protein content of tumor cells. In this paper, we demonstrate that tumor activated fibroblasts overexpress Galectin-1 (Gal-1) and consequently release MVs containing increased levels of this protein. The uptake of Gal-1 enriched MVs by tumor cells leads to the upregulation of its intracellular concentration, that strongly affects cancer cell migration, while neither proliferation nor adhesion are altered. Accordingly, tumor cells co-cultured with fibroblasts silenced for Gal-1 have a reduced migratory ability. The present work reveals the key role of an exogenous protein, Gal-1, derived from activated fibroblasts, in cancer progression, and contributes to clarify the importance of MVs-mediated protein trafficking in regulating tumor-stroma crosstalk.

## Introduction

Tumor progression is not only dependent on cancer cell genetic and epigenetic alterations but it is strongly supported by the tumor reactive stroma (Valkenburg et al. 2018). Indeed, tumor mass is a complex network of cancer and stromal cells, of whose fibroblasts represent the main component. Upon stimulation by tumor cells, fibroblasts engage a trans-differentiation program converting them into their activated form, known as cancer-associated fibroblasts (CAFs) (Kalluri 2016). Remarkably, CAFs are involved in all the crucial steps of tumorigenesis, by sustaining neo-angiogenesis (Orimo et al. 2005), the extracellular matrix (ECM) remodelling (Liu et al. 2019; Barbazán and Matic Vignjevic 2019) and the epithelial-to-mesenchymal transition (EMT) (Giannoni et al. 2010; Yu et al. 2014; Labernadie et al. 2017), thus promoting tumor cell migration and invasion, cancer stem cell (CSC) maintenance and metastatic dissemination (Santi et al. 2018). CAFs act also as a source of immunosuppressive molecules, thereby contributing to tumor immune escape (De Jaeghere et al. 2019). In addition, reciprocal metabolic symbiosis between CAFs and cancer cells supports invasive and drug resistant phenotypes in tumor cells and impairs antitumor immune responses, by altering the overall nutrient composition within the tumor microenvironment (TME) (Morandi et al. 2016; Comito et al. 2020).

The tumor-stroma crosstalk is mainly mediated by soluble paracrine factors, cell–cell contacts and extracellular vesicles (EVs) trafficking (van Niel et al. 2018). Interestingly, EVs trafficking acts either in an autocrine or paracrine manner within the TME, thereby representing a key form of intercellular communication.

Although EVs comprise a great heterogeneous population of membrane vesicles, to date two types of EVs, with different size, biogenesis and composition, have been described: microvesicles (MVs)/ectosomes (diameter from 100 nm to 1 μm) and exosomes (diameter from 30 to 100 nm) (Cocucci and Meldolesi 2015). With regard to EV biogenesis, exosomes are intraluminal vesicles (ILVs), that originate from inward budding of endosomal membrane during the multivesicular endosome (MVE) maturation process, and are secreted as a consequence of MVEs fusion with cell surface. Conversely, MVs are generated by the outward budding and fission of the plasma membrane and are then released in the extracellular space. EVs usually contain various cargoes, including proteins, lipids, and nucleic acids (DNA, mRNAs and miRNAs). The specific composition of the EV content strictly depends on the cell type, the stimuli and molecular mechanisms regulating their biogenesis and release, and the physio-pathological state of the donor cells. Once released in the extracellular space, EVs target recipient cells and deliver their content which then alters the phenotypical and functional properties of the targeted cells (van Niel et al. 2018).

Several recent findings underlined that CAFs are able to transfer EVs to tumor cells, providing significant evidence of EV-mediated back and forth exchange of factors between cancer and stromal cells within the TME (Shoucair et al. 2020). It is noteworthy that the uptake of CAF-derived EVs, usually loaded with specific miRNAs, lncRNAs and proteins, by recipient tumor cells strongly supports invasion and metastasis (Josson et al. 2015; Ren et al. 2018; Miki et al. 2018; Sun et al. 2019), and increases the proportion of CSCs (Hu et al. 2015). In addition, it has been demonstrated that patient-derived CAFs induce metabolic reprogramming of prostate and pancreatic tumor cells following EV uptake, ultimately promoting cancer cell growth. In particular, CAF-derived EVs supply nutrient-deprived tumor cells with various metabolites, including amino acids, lipids and tricarboxylic acid (TCA) cycle intermediates, to upregulate their central carbon metabolism (Zhao et al. 2016). Moreover, recent findings underscored that miR-21, transferred from CAFs to recipient ovarian tumor cells through exosomes trafficking, suppresses tumor cell apoptosis and confers paclitaxel resistance (Au Yeung et al. 2016). Similarly, exosome-mediated transfer of miR-92a-3p from CAFs to colorectal cancer cells promotes metastasis and 5-fluorouracil (5-FU)/ oxaliplatin (L-OHP) resistance (Hu et al. 2019).

Interestingly, a previous paper from our lab demonstrated that MVs transfer proteins and lipids essentially in a unidirectional way, from CAFs to cancer cells. In particular, MV components have been found involved in increasing melanoma and prostate cancer cell proliferation and in inducing the reverse-Warburg phenotype in recipient tumor cells. Several CAFs proteins specifically transferred to cancer cells by this type of vehicle have been also identified. Among them, Galectin-1 (Gal-1) emerged as one of the most enriched protein in CAF-derived EVs (Santi et al. 2015).

Gal-1 is the best characterized member of the galectin family (Johannes et al. 2018). Galectins are a phylogenetically conserved family of lectins and they are composed by amino acid sequences of about 130 amino acids with the carbohydrate recognition domain responsible for β-galactoside binding (Barondes et al. 1994). Gal-1 is generally localized in cell nuclei and cytoplasm, but it also translocates to the intracellular and extracellular side of cell membranes. Indeed, it displays the characteristics of typical cytoplasmic proteins, as well as an acetylated N-terminus and lack of glycosylation. It is noteworthy that Gal-1 can also be secreted in the extracellular matrices of various normal and neoplastic tissues (Cooper and Barondes 1990). Extracellular Gal-1 has been found altered in many cancer cell types (Thijssen et al. 2015), including melanoma (Yazawa et al. 2015), ovarian (Zhang et al. 2014) and prostate cancers (Laderach et al. 2013). Moreover, Gal-1 is often overexpressed in the reactive stromal cells in the TME (Valach et al. 2012). Increased expression of Gal-1 correlates with a variety of processes in cancer progression, including the cellular aggregation/tumor formation, cancer metastatic spread, angiogenesis, and apoptosis (Liu and Rabinovich 2005; Cousin and Cloninger 2016; Orozco et al. 2018).

In the present work, we reveal that the intercellular transport of Gal-1 from CAFs to tumor cells, mediated by MVs trafficking, leads to the upregulation of its steady state concentration in the recipient cancer cells and contributes to increase their migratory ability.

## Materials and methods

### Materials

Unless otherwise specified all reagents are from Sigma-Aldrich. Opti-MEM, FluoroBrite™ DMEM, Lipofectamine® transfection reagent, DAPI, CellTrace™ CFSE Cell Proliferation Kit and CellTracker™ Orange Dye are from Invitrogen™ Life Technologies. siRNAs for Gal-1, RNase-free DNase set are from Qiagen. Sh-RNA vectors are purchased from OriGene. RNA Nano Chip kit is from Agilent. PrimeScript RT reagent kit, ProteaseMAX™ Surfactant, Sequencing Grade Modified Trypsin are from Promega. Polyvinylidene difluoride (PVDF) membrane is from Millipore. Transwells are purchased from Euroclone. Anti-Gal-1 antibody is from Cell Signaling, anti-β-actin and anti-Integrin-β1 antibodies are from Santa Cruz Biotechnology, anti-CD81 antibody is from BD Biosciences. The secondary antibodies enzyme horseradish peroxidase (HRP)–conjugated are from Santa Cruz Biotechnology. All materials for SDS-PAGE and Clarity™ Western ECL substrate are from Biorad. Culture-inserts (Dish 35 mm, high) are from ibidi®.

### Cell cultures

Human prostate (DU145 and LNCaP), pancreatic (PANC-1), and melanoma (A375) cancer cells were purchased from European Collection of Cell Culture (ECACC). Normal Human Fibroblasts (NHFs) used in our experiments were Human Dermal Fibroblasts (HDFs, from Invitrogen™ Life Technologies) and Human Prostate Fibroblasts (HPFs). HPFs were isolated from surgical explantation of patients who signed informed consent in accordance with the Ethics Committee of Azienda Ospedaliera Universitaria Careggi by Prof. Serni of Dipartimento di Medicina Sperimentale e Clinica/Urology (Firenze, Italy) (Giannoni et al. 2010). No significant differences were actually determined in the behavior of the two fibroblast lines. All cells were routinely cultured in Dulbecco's Modified Eagle's Medium (DMEM)—high glucose (4500 mg/L), except for LNCaP cells that were cultured in RPMI medium. Both media were supplemented with 10% fetal bovine serum (FBS, Euroclone), 2 mM glutamine, 100 U/mL penicillin and 100 μg/mL streptomycin. Cells were incubated at 37 °C in a humidified atmosphere of 5% CO_2_.

### Conditioned media preparation and fibroblast activation

Tumor cells (DU145, A375, PANC-1) were incubated in growth medium with 1% of serum depleted by MVs and exosomes (EVs depleted FBS) for 24 h. Serum depletion was performed by centrifugation at 10.000×g for 1 h and subsequently at 100.000 × g for 90 min. After 24 h of incubation with tumor cells the medium was recovered, centrifuged at 300 xg for 20 min to discard cell debris and used to culture fibroblasts for 24 h, obtaining their activated forms (A-HDFs and A-HPFs), superimposable to native CAFs (Giannoni et al. 2010).

### Purification of membrane vesicles secreted by fibroblasts

Normal Human Fibroblasts (NHFs: HPFs or HDFs) or Activated Human Fibroblasts (AHFs: A-HPFs or A-HDFs) were cultured for 24 h in growth medium supplemented with 1% EVs depleted FBS. To isolate the MVs fraction, the medium was recovered and centrifuged at 300 xg for 10 min to discard cells, at 2000 × g for 20 min to discard cell debris and finally at 10.000 × g for 1 h to pellet the MVs fraction. The pellet was resuspended in PBS and centrifuged again at 10.000 × g for 1 h obtaining a purified MVs fraction. To isolate the exosomes fraction, the supernatant recovered after the 10.000 × g centrifugation was further centrifuged at 100.000 × g for 1 h and the pellet resuspended in PBS (Xu et al. 2015).

### Western blot analysis

For electrophoresis and western blot (WB) analysis, MVs, exosomes or cell lysates were suspended in twofold concentrated Laemmli electrophoresis buffer (without β-mercaptoethanol and bromophenol blue) and assayed for protein content by the BCA method. Equal amount of total protein (20–40 μg) from each sample were additioned with β-mercaptoethanol and separated by SDS-PAGE. Subsequently, gels were electroblotted onto PVDF membranes. The blots were incubated with the selected primary antibody in order to evaluate specific protein content as indicated in the figures. After incubation with secondary antibodies, the blotting was developed by using the ECL plus immunodetection system.

### Quantitative real time polymerase chain reaction (qPCR)

Quantitative PCR reactions were performed by using GoTaq qPCR master mix kit and the Mx3005P Stratagene system. Differential expressions of transcripts of interest were calculated in relation to the h36B4 housekeeping transcript for cDNA. The primers for GAL-1 were:

- Forward: 5’-TCGCCAGCAACCTGAATCTC-3’ Reverse: 5’-GCACGAAGCTCTTAGCGTCA-3.

### Transwell co-culture system

Co-culture experiments were performed by using Transwell permeable supports with pore sizes of 0.4 µm or 8 µm. Tumor cells were seeded in 6-well plates, while NHFs were plated on the upper compartment of the Transwells in a 2:1 ratio. After 24 h of incubation, protein content of cancer cells was analysed by western blot.

To verify that fibroblasts, seeded in the upper side of the 8 µm Transwells, do not traverse the filter and are not collected with tumor cells, NHFs were stained with 10 µM CFDA-SE in Hank’s Balanced Salt Solution (HBSS) for 15 min at 37 °C. Then, NHFs were incubated with complete DMEM for 1 h. After that, NHFs were harvested by trypsinization. Fibroblasts and tumor cells were co-cultured by using Transwell permeable supports with pore size of 8.0 µm. Specifically, fibroblasts were seeded in the upper side of the insert, while tumor cells were seeded in the lower compartment. After 24 h of incubation, cells in the lower compartment of the Transwell system were harvested by trypsinization, washed in PBS, and fixed in 3% paraformaldehyde. CFDA-SE fluorescence was analysed by flow cytometry. Tumor cells not co-cultured with NHFs were used as negative control. CFDA-SE-stained NHFs not co-cultured with cancer cells were used as positive control.

### siRNA cell transfection

Silencing with siRNA plasmid was performed with Lipofectamine™ 2000, following manufacturer’s instructions. Cells were plated at 70% confluence and, before transfection, DMEM culture medium was removed and replaced with Optimem medium, lacking serum and antibiotics that could interfere with liposome formation. Solution containing siRNA was added to solution with Lipofectamine and incubated at room temperature for 20 min, in order to promote liposome formation; then equal amounts of final solution were added to each plate. Optimem medium was removed after 4–6 h from transfection, as Lipofectamine could be slightly toxic for cells. Finally, cells were maintained in complete medium for 48 h and transfection efficiency was evaluated through immunoblotting assays. Gal-1 expression was silenced using four different pre-designed siRNAs directed against different regions of the Gal-1 transcript (Qiagen, Hilden, **siRNA3** = Hs_LGALS1_3 HP, Cat. No. SI00035924; **siRNA5** = Hs_LGALS1_5 Cat. No. HP SI02628269; **siRNA6** = Hs_LGALS1_6 HP, Cat. No. SI03033947; **siRNA7** = Hs_LGALS1_7 HP, Cat. No. SI03085453). siRNA7 was more effective in terms of down-regulating Gal-1 expression on protein level compared to the others. The morphology of control and Gal-1 silenced DU145 cells was evaluated by taking photographs at randomly chosen fields using the inverted microscope Nikon Eclipse TS100.

### Sh-RNA cell transfection

Lentiviral particles were generated by transfecting 293 T cells with a mix of 1/3 pGFP-C-shLenti construct (4 different vectors for Gal-1), 1/3 delta Helper (carries sequence necessary for viral assembly of lentivirus) and 1/3 pVsVg (expresses the vesicular stomatitis virus envelop glycoprotein G pseudotype), using Lipofectamine™ 3000 Reagent and following manufacturer’s recommendations. 24 h post transfection, the medium was changed for fresh one. 24 h later, medium was changed again and viruses containing medium was collected, filtered through a 0.2 μm filter, and added on 40% confluent PANC-1 and NHFs cells seeded in 6 multi-well plates. This step was repeated 24 h later to perform a second infection. Five days after infection, expression of Green Fluorescent Protein (GFP) was verified by fluorescence microscopy and Gal-1 expression was evaluated by WB analysis. The vectors used were:

- #A AACCTGGAGGCCATCAACTACATGGCAGC
- #B TCTGGTCGCCAGCAACCTGAATCTCAAAC
- #C GACGGTGACTTCAAGATCAAATGTGTGGC
- #D CCTTCCAGCCTGGAAGTGTTGCAGAGGTG

The higher silencing has been found with vectors #A and #B for both PANC-1 and NHFs. Results referred to PANC-1 and NHF cells silenced with vector #A.

The morphology of control and Gal-1 silenced PANC-1 cells was evaluated by taking photographs at randomly chosen fields using the inverted microscope Nikon Eclipse TS100.

### Proliferation assay

The proliferation of DU145 cells was evaluated using CFDA-SE probe. Tumor cells, control or silenced for Gal-1, were labeled with the dye at the concentration of 2.5 μM. Then cells were cultured and after 24 and 48 h were detached, fixed in 3% paraformaldehyde and analysed by flow cytometry (BD FACSCanto II, BD Biosciences-US). The obtained fluorescence value was analysed by ModFit software to estimate the proliferation index.

The proliferation of GFP positive PANC-1 cells, control and silenced for Gal-1, was assessed by counting cells with Bürker’s chamber three times per condition 24 and 48 h after plating.

### Adhesion assay

2 × 10^5^ cells were seeded in 35 mm dishes. After 0.5, 1, 2 and 3 h, the number of adherent tumor cells was evaluated with crystal violet staining for 5 min at 37 °C. Fixed cells were washed with PBS and solubilized with 0,1 M Sodium Citrate, pH 4,2. The absorbance was evaluated at 595 nm.

### Migration assay

PANC-1 migration was evaluated using Transwell systems, equipped with 8 μm pore polyvinylpirrolidone-free polycarbonate filters (6.5 mm diameter), coated with 1% fibronectin in 0.1% gelatin and stored at 4 °C. DU145 migration was evaluated with the same Transwell system but without coating. 5 × 10^4^ cells resuspended in 150 μL of DMEM with 1% EVs depleted FBS were seeded in the upper compartment of Transwells placed into 24-well culture dishes. In the lower compartment, 500 μl of DMEM with 1% EVs depleted FBS for PANC-1 cells and DMEM with 10% EVs depleted FBS for DU145 cells was added. Then, PANC-1 and DU145 control cells were treated with 50 μL of DMEM with 1% EVs depleted FBS. Besides, tumor cells were treated with MVs isolated from normal fibroblasts (HDFs or HPFs) or from activated fibroblasts (A-HDFs or A-HPFs). Specifically, isolated MVs were resuspended in 50 μL of DMEM with 1% EVs depleted FBS and then added to cell medium in the upper compartment of the Transwells. After 6 h for PANC-1 cells and 16 h for DU145 cells, while the insert was still moist, the non-migratory cells were mechanically removed from the interior of the insert using a cotton swab. Migrated cells were fixed in cold methanol, labeled with DAPI and photographed by fluorescence microscopy (Leica TCS SP5). Chemotaxis was evaluated by counting the cells migrated to the lower surface of the filters as a mean of twenty randomly chosen fields.

### Wound healing assay

PANC-1 and DU-145 cells were labeled with 5 μM CellTracker™ Orange Dye and co-cultured with GFP-NHF cells (control and silenced for Gal-1) in Dish^35mm,high^ Culture-insert in ratio 1:1. The medium used for the co-culture was the FluoroBrite™ DMEM that does not interfere with fluorescence. The day after the insert was removed and pictures at time 0 and 24 h were acquired with Leica FX 350 camera in a Leica AM 600 microscope, using these parameters: L5: ex BP 480/40, dichroic 505, em BP 527–30; N2.1 ex BP 515/560, dichroic 565, em LP 590.

### Statistical analysis

Data are presented in bar graphs as means ± SD from at least three independent experiments (unless specified). Statistical analysis of the data was performed by Student’s */t/*-test or ANOVA followed by Tukey HSD test. */p/*-values of ≤ 0.05 were considered statistically significant. Single asterisk indicates a significant difference at */p/*-value < 0.05, double asterisks indicate a significant difference at */p/*-value < 0.01 and triple asterisks indicate a significant difference at */p/*value < 0.001.

## Results

### Gal-1 is upregulated upon fibroblast activation and transferred to cancer cells using MVs as vehicles

The interplay between fibroblasts and cancer cells within the TME induces remarkable changes in the phenotypic features of both cell types, favoring cancer progression (Kalluri 2016). In particular, upon stimulation with pro-inflammatory cytokines secreted by cancer cells, fibroblasts trans-differentiate in their activated form (CAFs) (Giannoni et al. 2010). One of the most important consequences of fibroblast activation is the increase in their ability to produce and secrete MVs, which are then uploaded in cancer cells, thereby playing an essential role in sustaining their proliferation. We previously identified various proteins that are specifically transferred from CAFs to cancer cells using MVs as vehicle (Santi et al. 2015). Here we focused our attention on one of these proteins, Gal-1, that is both one of the most present proteins in CAF-derived MVs and a protein known to be involved in many aspects of cancer progression (Liu and Rabinovich 2005).

**Fig. 1:**
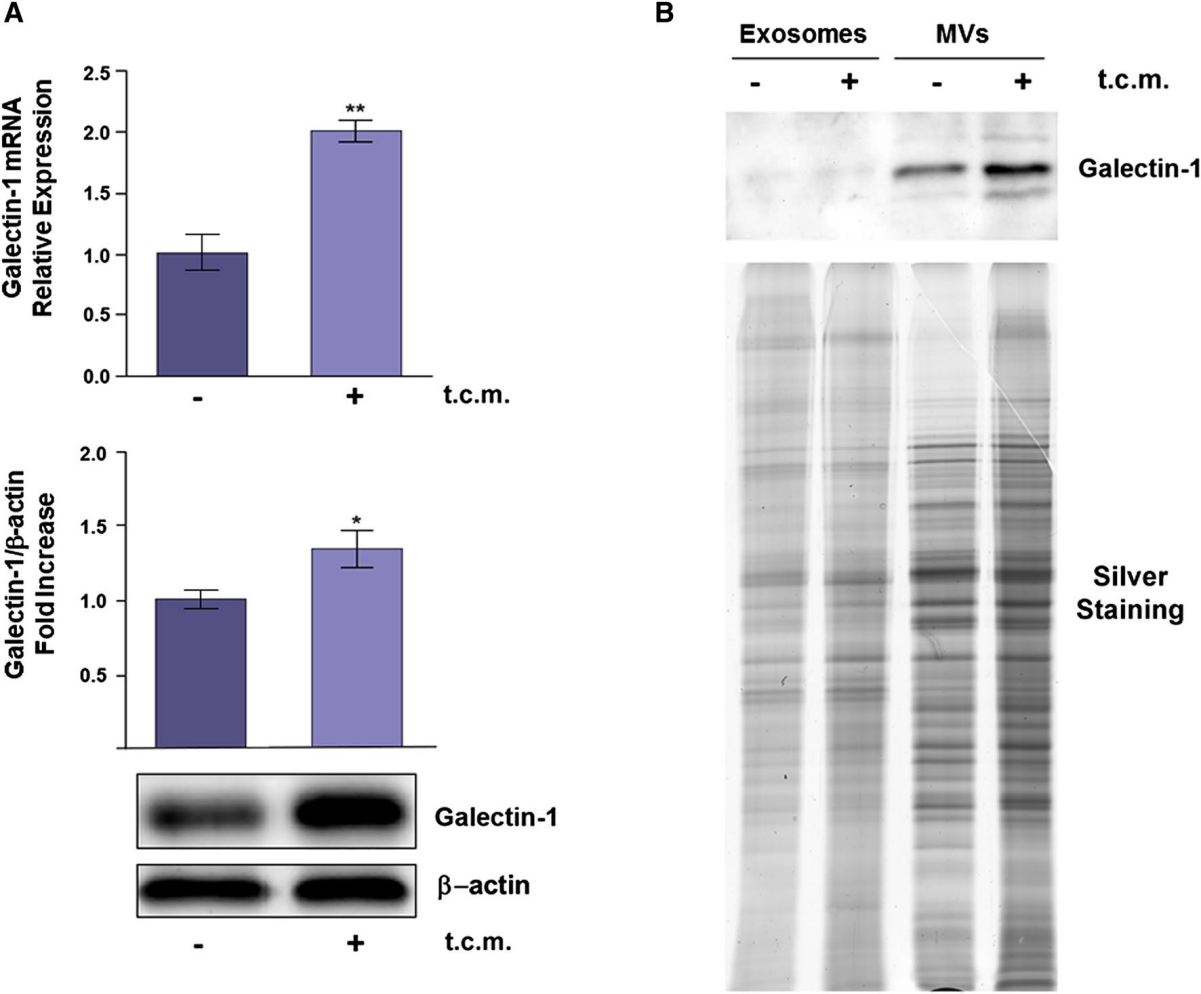
Gal-1 is overexpressed in activated fibroblasts and released into the extracellular milieu using MVs as vehicles. **A** Normal Human Fibroblasts (NHFs) were treated with t.c.m. from tumor cells for 24 h. qRT-PCR and WB analyses were performed to evaluate Gal-1 mRNA and protein expression levels, respectively, in t.c.m. activated NHFs (AHFs) with respect to NHFs. Data shown represent mean + / − SD from three independent experiments (*p < 0.05; **p < 0.01). **B** NHFs and AHFs were incubated for 24 h with fresh DMEM supplemented with 1% EVs depleted FBS. Then*,* MVs and exosomes from NHFs and AHFs were isolated through differential centrifugation (see “Materials and methods”) and their total protein content analysed by SDS-PAGE and silver staining. The Gal-1 content was evaluated by WB with anti-Gal-1 antibody. 30 µg aliquots of proteins were loaded for each sample. The data shown are representative of at least three experiments with similar results

Firstly, we found that NHFs upregulate both Gal-1 mRNA and protein levels when treated for 24 h with tumor conditioned medium (t.c.m.) from tumor cells (Fig. 1A). Treatment with t.c.m. converts normal fibroblasts into their activated forms (AHFs), that express the same protein markers and share the same cellular properties of native CAFs isolated from tumors (Giannoni et al. 2010; Santi et al. 2015; Hu and Hu 2019).

All these experiments were performed using both Human Prostate Fibroblasts (HPFs) and Human Dermal Fibroblasts (HDFs), obtaining similar results. We then analysed the Gal-1 expression levels in EVs, i.e. MVs and exosomes, synthesized and secreted by normal and activated fibroblasts (Fig. 1B). Figure 1B clearly shows that Gal-1 is mostly present in MVs respect to exosomes and its expression in MVs is increased upon fibroblast activation.

Overall, our data highlight that tumor secretome induces Gal-1 overexpression in activated fibroblasts as well as in their secreted MVs.

In order to dissect the mechanism of Gal-1 transfer from activated fibroblasts to cancer cells, we used Transwell systems with different pore sizes (0.4 or 8 μm). In this kind of co-culture, we observe the rapid activation, within few hours (data not shown), of fibroblasts into their activated counterpart due to cytokine-mediated crosstalk. Activated fibroblasts, in turn, increase their overall release of MVs containing Gal-1.

**Fig. 2:**
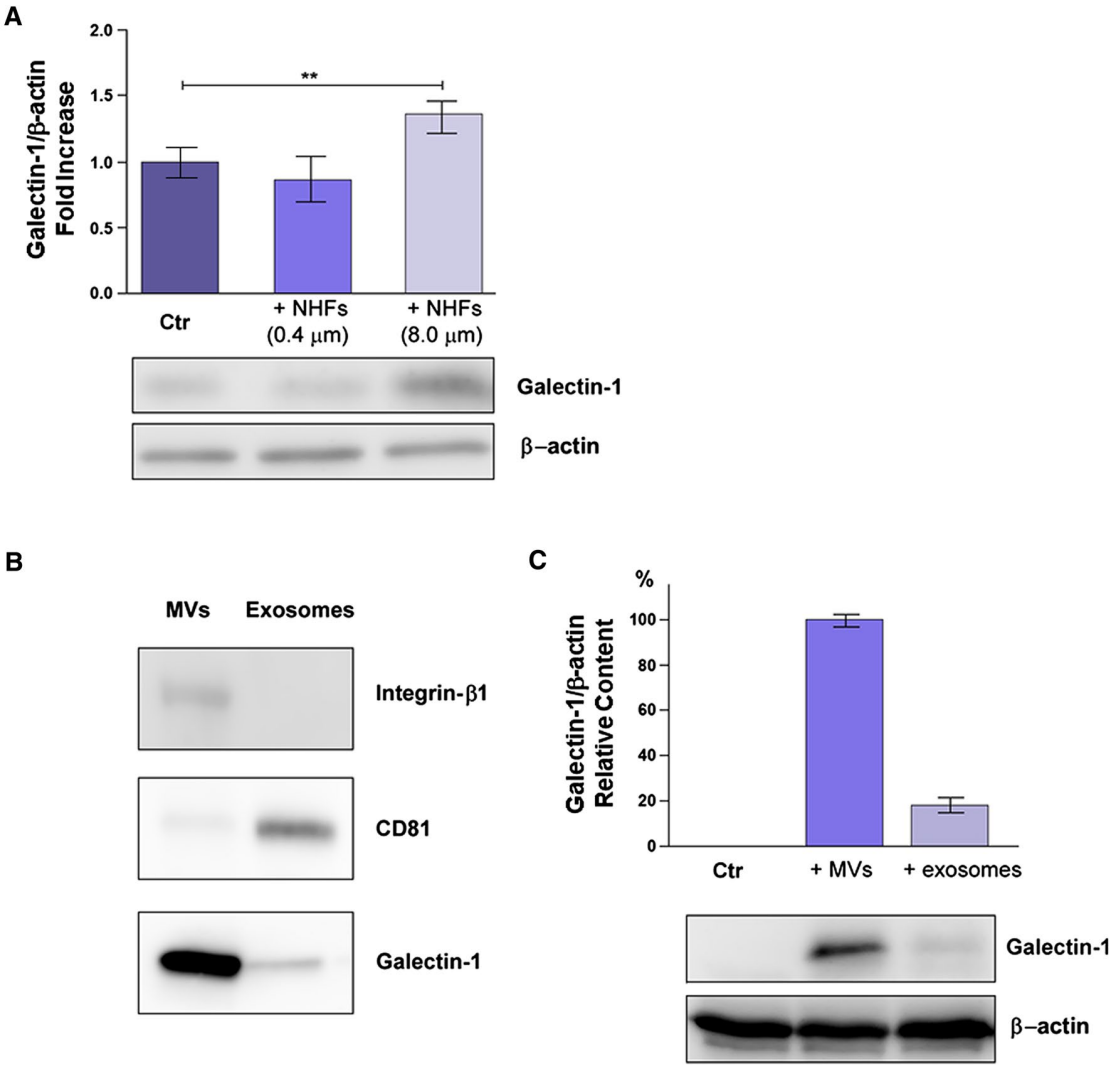
Gal-1 is transferred to tumor cells using MVs as vehicles. **A** DU145 tumor prostate cells were seeded in a 6-well plate, while fibroblasts (NHFs) were plated on Transwells of different pore sizes (0.4 μm or 8 μm). The co-culture rapidly induces the fibroblasts activation. After 24 h cancer cells in the lower chamber were lysed and subjected to WB analysis. Quantification plot of Gal-1 expression, normalized using β-actin, was reported as fold increase respect to control (cancer cells in the lower chamber without fibroblasts plated on the filter). Data represent mean + / − SD from four independent experiments (**p < 0.01). **B** NHFs were activated for 24 h with t.c.m. Then, fresh growth medium with 1% FBS (depleted of EVs) was added for the following 24 h in order to collect fibroblast-derived MVs and exosomes. WB of AHF-derived MVs and exosomes, quantified for protein content, was performed to characterize specific markers of MVs (integrin-β1) and exosomes (CD81). Equal amounts (20 µg) of each sample were analysed. **C** LNCaP cells were treated for 4 h with purified MVs or exosomes from A-HPF, resuspended in growth medium with 1% EVs depleted FBS. Then, the amount of Gal-1 in LNCaP cells was evaluated by WB analysis. The data shown are representative of at least three experiments with similar results

Figure 2A shows that only when fibroblasts and DU145 cancer cells were co-cultured in 8 μm Transwells we observed an upregulation of Gal-1 in recipient tumor cells. Hence, Gal-1 is not transferred by exosomes, that easily pass through 0.4 μm filters, nor Gal-1 derives from a de novo synthesis in cancer cells induced by fibroblast-derived cytokines for the same reason. In addition, we found that NHFs are not able to pass the 8.0 μm filters and adhere to the lower compartment of Transwell systems where tumor cells are seeded, indicating that analysed cell lysates were specifically obtained from tumor cells. Overall these data underline that Gal-1 is transferred exclusively via MVs trafficking from fibroblasts to tumor cells. Analogous experiments were performed on other tumor cell lines obtaining similar results (data not shown).

Finally we purified and characterized MVs and exosomes from AHFs using integrin-β1 and CD81 as specific markers of MVs and exosomes, respectively (Fig. 2B). Purified MVs and exosomes were then incubated for 4 h with LNCaP cells, a prostate cancer cell line characterized by extremely low expression levels of Gal-1. Interestingly, Gal-1 expression levels are strongly increased in cell lysates from LNCaP cells treated with MVs, but only at a lower extent in tumor cells incubated with purified exosomes (Fig. 2C).

### Exogenous Gal-1 improves tumor cell migration

**Fig. 3:**
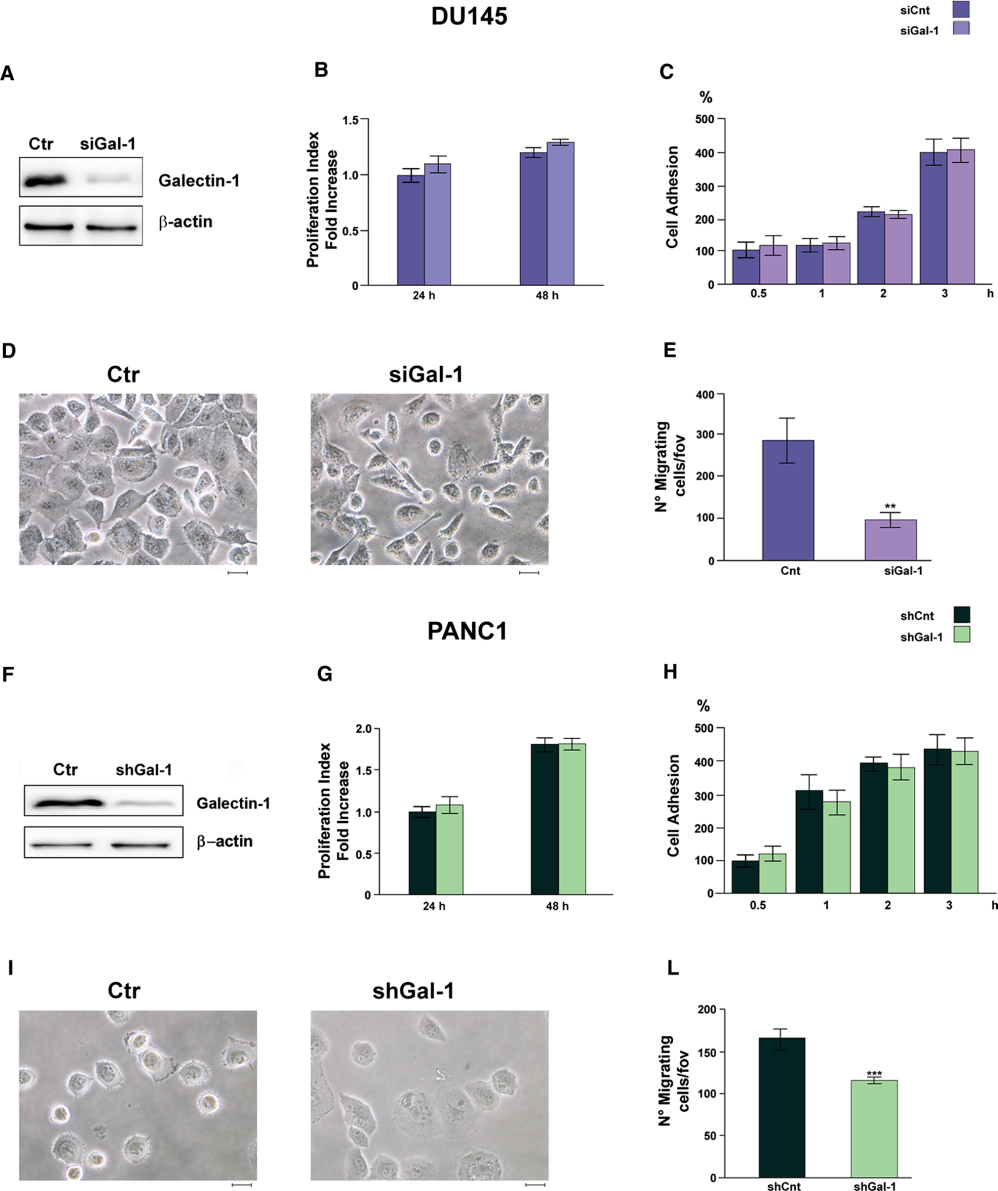
Gal-1 enhances cell migration but not proliferation nor adhesion of DU145 and PANC-1 cells. Cell proliferation, cell adhesion, morphology and cell migration were evaluated on DU145 and PANC-1 cells silenced or not for Gal-1 expression. **A** WB analysis of Gal-1 expression of control and silenced DU145 cells. Silencing has been achieved by siRNA (see “Materials and methods”). **B** Proliferation index of control and silenced DU145 cells using CFDA-SE assay (see “Materials and methods”). **C** Cell adhesion assay of control and silenced DU145 cells. **D** Cell morphology of control and Gal-1 silenced DU145 cells. Scale bar = 50 µm. **E** Migration assay of control and silenced DU145 cells. **F** WB analysis of Gal-1 expression of control and silenced PANC-1 cells. Silencing has been achieved by shRNA (see “Materials and methods”). **G** Proliferation index of control and silenced PANC-1 cells using Burker’s chamber cell count. **H** Cell adhesion assay of control and silenced PANC-1 cells. **I** Cell morphology of control and Gal-1 silenced PANC-1 cells. Scale bar = 50 µm. **L** Migration assay of control and silenced PANC-1 cells. For all kind of tests data represent mean + / − SD from at least three independent experiments (**p < 0.01; ***p < 0.001)

Gal-1 is widely reported as a protein able to influence several aspects of tumor progression (Cousin and Cloninger 2016). In order to assess its role in our experimental setting, we silenced Gal-1 expression in both DU145 and PANC-1 cells (Fig. 3A, F).

Gal-1 silenced cells do not show differences nor in the cell proliferation rate (Fig. 3B, G) or in the adhesion properties (Fig. 3C, H), with respect to control cells. By contrast, Gal-1 silenced DU145 and PANC-1 cells exhibit distinct morphological traits when compared to their not-silenced counterpart. In particular, control cells display a higher number of cellular protrusion (Fig. 3D, I), that results in increased migratory potential. Indeed, Gal-1 silencing strongly impairs both DU145 and PANC-1 migratory ability (Fig. 3E, L).

**Fig. 4:**
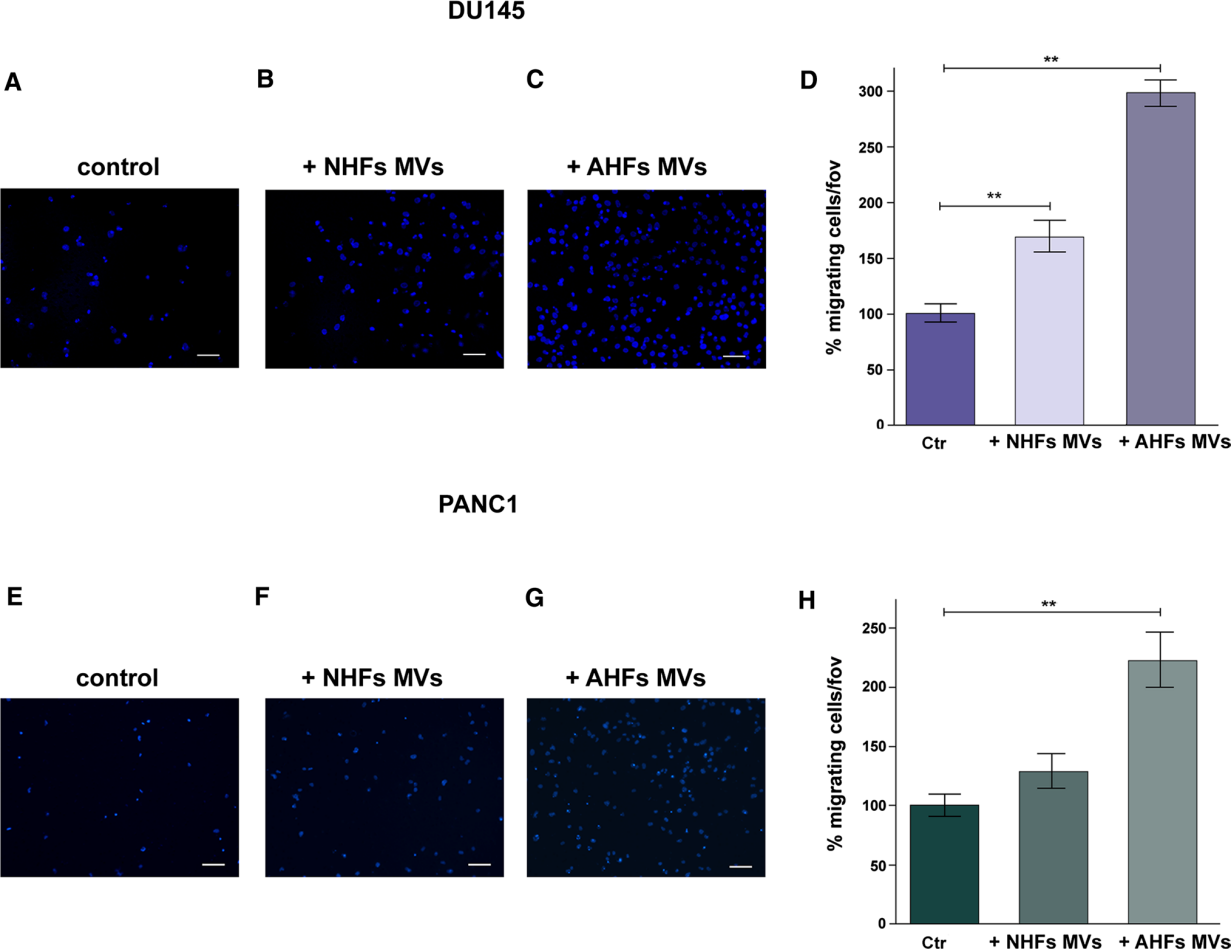
DU145 and PANC-1 migration is strongly enhanced by MVs derived from activated fibroblasts. Boyden chamber migration assay was performed on DU145 and PANC-1 cells treated or not with MVs derived from both normal fibroblasts and activated fibroblasts. **A**–**C** DU145 cells cultured in the presence of: **A** DMEM supplemented with 1% FBS depleted of EVs (control); **B** MVs purified from NHFs; **C** MVs purified from AHFs (see “Materials and methods”). **D** Quantification of DU145 cell migratory capacity expressed as fold increase of migrated cells respect to control. **E–G** PANC-1 cells cultured in the presence of: **E** DMEM supplemented with 1% FBS depleted of EVs (control); **F** MVs purified from NHFs; **G** MVs purified from AHFs (see “Materials and methods”). **H** Quantification of PANC-1 cell migratory capacity expressed as fold increase of migrated cells respect to control. Scale bar = 50 µm. Data represent mean + / − SD from three independent experiments (**p < 0.01)

Moreover we observed that purified MVs derived from AHFs (Fig. 4C, G) highly enhance both DU145 and PANC-1 migration when compared to control cells treated with DMEM with 1% FBS depleted of EVs (Fig. 4A, E). Interestingly, MVs purified from NHFs are less efficient in promoting DU145 and PANC-1 cell migration respectively (Fig. 4B, F).

**Fig. 5:**
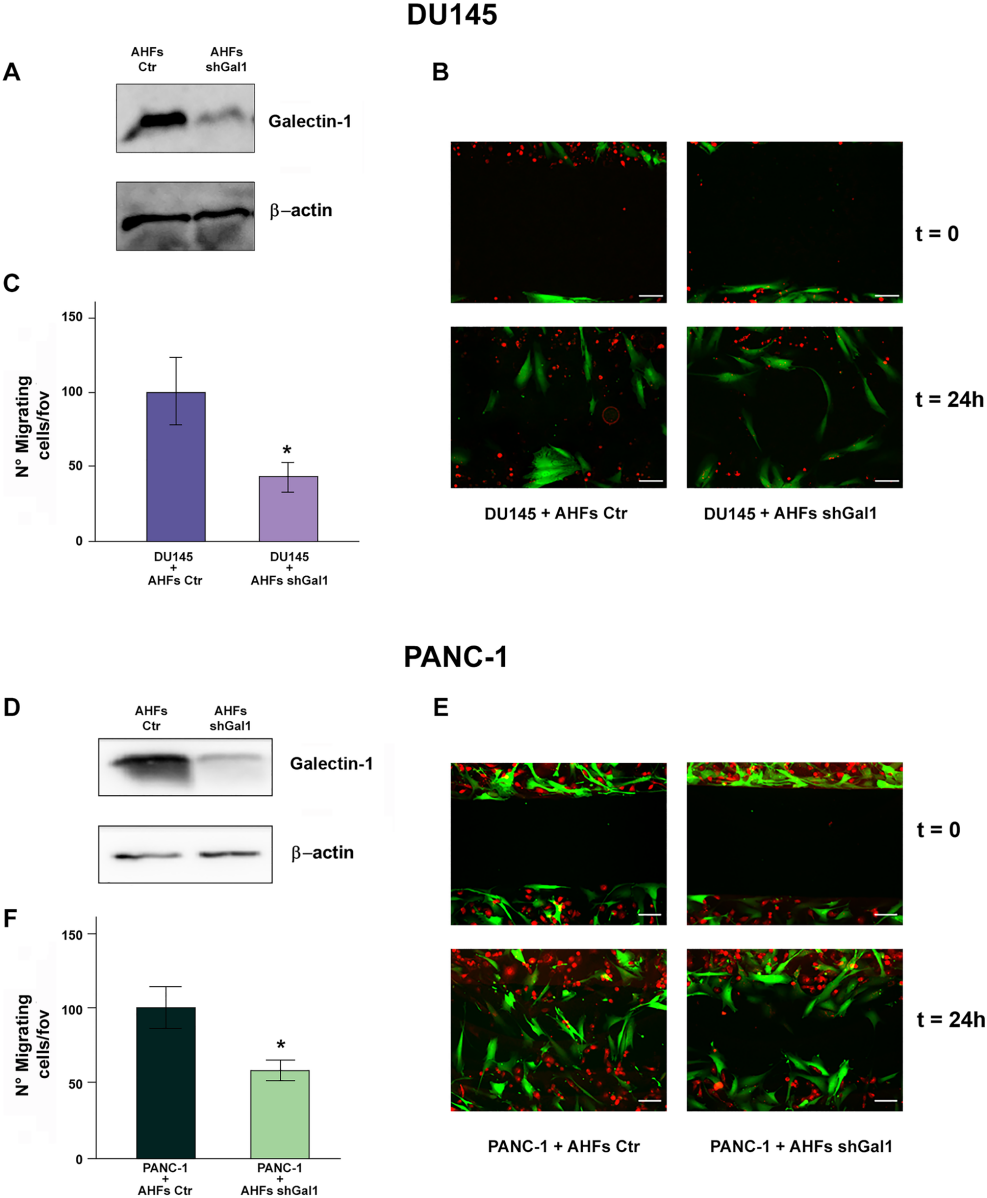
Exogenous Gal-1 derived by activated fibroblasts promotes migration in tumor cells. **A**, **D** Stable Gal-1 silencing in AHFs was evaluated by WB analysis. β-actin was used for normalization. **B** Wound healing assay of DU145 cells (labeled with Cell Tracker™ Orange Dye) in co-culture with AHFs (GFP positive) silenced or not for Gal-1 expression. Scale bar 200 µm. **C** Mean of DU145 cells present in the wound after 24 h, without considering cells placed at a distance of less than 50 μm from both sides of the scratch. **E** Wound healing assay of PANC-1 cells (labeled with Cell Tracker™ Orange Dye) in co-culture with AHFs (GFP positive) silenced or not for Gal-1 expression. Scale bar 200 µm. **F** Mean of PANC-1 cells present in the wound after 24 h, without considering cells placed at a distance of less than 50 μm from both sides of the scratch. Data represent mean + / − SD from three independent experiments (*p < 0.05)

To demonstrate that fibroblast-derived Gal-1 is directly implicated in tumor cell migration, we set up a wound healing assay in which PANC-1 and DU145 cells, labeled with CellTracker™ Orange Dye, were seeded in co-culture with AHFs, expressing GFP and stably silenced or not for Gal-1 (Fig. 5A, D). PANC-1 and DU145 cells co-cultured with control AHFs result to have a greater migratory ability compared to cancer cells co-cultured with Gal-1 silenced AHFs (Fig. 5B, E). To quantify the results of this experiment, PANC-1 and DU145 cells present in the scratch at 24 h were counted, excluding those placed at a distance less than 50 μm from the boundary of the scratch, to avoid considering cancer cells movement due to proliferation (Fig. 5C, F).

These findings evidence a novel mechanism exerted by CAFs to support tumor invasiveness through exogenous protein transfer via MVs.

## Discussion

Solid tumors are composed by cancer cells and tumor reactive stroma (Wang et al. 2017). In the last decades the key role of stromal cells in promoting cancer aggressiveness has been the focus of several studies. Fibroblasts are the major stromal component in the TME and, in this context, they switch into their activated phenotype, namely CAFs. Cancer progression is strongly influenced by CAFs, that support ECM remodeling, angiogenesis, and inflammatory cells recruitment, via secretion of cytokines, chemokines, growth factors and EVs (Kalluri 2016; Barbazán and Matic Vignjevic 2019; Choe et al. 2013).

Santi and co-workers recently discovered a horizontal unidirectional transfer of proteins and lipids from CAFs to cancer cells, mostly mediated by MVs. They demonstrated that, even if structural proteins and glycolytic enzymes represent the 70% of total mass transferred from CAFs to cancer cells, the majority of these proteins are not enriched in tumor cells. By contrast, among the CAF-derived proteins specifically upregulated in recipient cells through MVs trafficking, Gal-1 resulted to be one of the most represented (Santi et al. 2015). Therefore, in this paper we focus our attention on MV-mediated transfer of Gal-1 from CAFs to tumor cells in affecting the migratory abilities of recipient cancer cells.

Several studies investigated the role of stromal Gal-1 upregulation in supporting tumor progression and aggressiveness, thus revealing its possible application as a novel therapeutic target for many types of cancer. In particular, Gal-1 has been found overexpressed in cancer-associated stromal cells of gastric adenocarcinoma and breast and prostate tumors, correlating with increased tumor invasiveness and metastasis (van den Brûle et al. 2001; Jung et al. 2007; Bektas et al. 2010). Moreover, Tang and co-workers reported that CAF-derived Gal-1 strongly promotes angiogenesis in gastric cancer by sustaining endothelial cell proliferation, migration and tube formation (Tang et al. 2016). However, the molecular mechanisms by which high levels of exogenous Gal-1 in the stromal compartment affect cancer cell aggressiveness have not yet been completely clarified. Interestingly, in vitro and in vivo studies on pancreatic ductal adenocarcinoma (PDAC) underlined that stromal Gal-1, that is highly overexpressed by stromal fibroblasts and pancreatic stellate cells, is directly secreted in the TME and consequently establishes paracrine crosstalk with epithelial tumor cells to further trigger proliferation and invasion of cancer cells, enhance angiogenesis and inhibit immune cell infiltration (Xue et al. 2011; Martínez-Bosch et al. 2014; Orozco et al. 2018).

In the present paper, we demonstrate that Gal-1 expression in various cancer cell types can be upregulated through its highly efficient transfer via-MVs from activated fibroblasts to tumor cells, rather than being directly secreted in the extracellular environment by stromal cells or being modulated by the more common intercellular signaling mediated by paracrine factors.

It is noteworthy that protein transfer via EVs from CAFs to cancer cells has not been largely addressed, since the majority of the studies focused on the role of EV-mediated transfer of miRNAs within the TME (Shoucair et al. 2020). Notably, we show that the conversion of normal fibroblasts into their activated counterparts, upon stimulation with cytokines in the tumor conditioned media from cancer cells, causes the upregulation of Gal-1 expression levels, both in their intracellular compartment and in their secreted MVs (Fig. 1). Indeed, the protein content of EVs is strictly dependent on the cell type they originate from, the biogenesis and the stimuli driving their release (Zaborowski et al. 2015).

Despite MVs trafficking is currently emerging as a critical mediator of intercellular communication in the context of the tumor-stroma crosstalk (Muralidharan-Chari et al. 2010; Menck et al. 2020), its function is still poor defined. For instance, most studies concerning EVs transfer during cancer progression focused on exosomes (Dai et al. 2020) or mixed vesicle populations and it is still largely unclear which sub-population of EVs is responsible for a given physio-pathological effect (van Niel et al. 2018; Han et al. 2019; Maacha et al. 2019). The reason for such a lack of information regarding MV trafficking is mainly due to the challenges encountered during the selective isolation of specific sub-species of vesicles. Indeed, although numerous reports have been published on comparative methods for EV isolation, including density gradient ultracentrifugation, size exclusion chromatography, precipitation via volume-excluding polymers, flow-cytometry, and high pressure liquid chromatography, we are still far from having pure isolation of exosomes and MVs (Konoshenko et al. 2018; Théry et al. 2018). In this context, we checked the purity of EV separation, obtained through differential centrifugation, by using Integrin β1 and CD81 as markers of MVs and exosomes, respectively (Fig. 2) (Santi et al. 2015).

Remarkably, we highlight that the specific intercellular trafficking of MVs from activated fibroblasts to cancer cells mediates the transfer of Gal-1 to the latter. In fact, when tumor cells are co-cultured with fibroblasts in Transwell systems with 0.4 μm pore size, that allow the free passage of exosomes and paracrine soluble factors released by fibroblasts but not the transfer of MVs, Gal-1 expression levels are not increased in cancer cells. Conversely, when using Transwell systems with 8 μm pore size, freely permeable to MVs, in tumor cells/fibroblasts co-culture settings, Gal-1 expression is upregulated in tumor recipient cells (Fig. 2). In addition, LNCaP cells, which are characterized by endogenous extremely low levels of Gal-1, treated with MVs purified from AHFs display an appreciable increase in the expression levels of Gal-1, when compared to cells treated with purified exosomes from AHFs (Fig. 2). These results further indicate that Gal-1 is transferred from activated fibroblasts to cancer cells specifically via MVs. This finding is consistent with our previous evidence highlighting that MVs are more efficient than exosomes in transferring proteins to recipient cells (Santi et al. 2015).

Along with Gal-1 upregulation in the tumor-associated stromal compartment, Gal-1 has been found overexpressed also in cancer cells. Indeed, it has been reported that the overexpression of this protein in different tumor types correlates with several processes of cancer malignancy, including tumor cell proliferation, migration, invasion and T cell activation (Spano et al. 2010; Kim et al. 2012; Noda et al. 2017), and it is associated with patient worse prognosis (Kim et al. 2013; Chen et al. 2014; Yazawa et al. 2015). Therefore, we evaluate the biological effects of Gal-1 upregulation in our cancer cell models. To this aim, we silenced Gal-1 expression in two different cancer cell lines: DU145, a prostatic cancer cell line, and PANC-1, a pancreatic adenocarcinoma cell line. This latter represents a kind of tumor characterized by a high degree of stromal infiltration and fibrosis (von Ahrens et al. 2017). In DU145 as well as in PANC-1 cells, Gal-1 downregulation does not affect cell proliferation nor cell adhesion rate, while strongly impairs cell migration (Fig. 3). Accordingly, it has been demonstrated that Gal-1 is highly overexpressed in castrate resistant prostate cancer (CRPC) and its knockdown significantly decreases prostate cancer cell migration and invasion (Shih et al. 2018). Similarly, Gal-1 plays a pivotal role in PDAC progression, by inducing tumor growth, immune evasion and angiogenesis (Berberat et al. 2001; Roda et al. 2009; Martinez-Bosch et al. 2018). Overall, besides the already described role of Gal-1 overexpression in the stromal compartment of pancreatic and prostate tumors (van den Brûle et al. 2001; Orozco et al. 2018), the upregulation of this protein in cancer cells is crucial for tumor malignancy.

MVs recently emerged as important vehicles for the transfer of bioactive molecules within the TME. Indeed, the deliver of MV cargoes in tumor cells strongly alters their functional characteristics. However, the majority of these studies investigated the effects of tumor-derived MVs in modulating the behavior of either tumor cells, via autologous cell–cell communication (Al-Nedawi et al. 2008; Arendt et al. 2014), or neighboring TME cells, including endothelial cells (Kawamoto et al. 2012), fibroblasts (Jiang et al. 2019) and immune cells (Baj-Krzyworzeka et al. 2007; Cui et al. 2018), through heterologous cell–cell communication. Conversely, the effects of CAF-derived MVs in cancer progression still need to be elucidated. Since Gal-1 plays a fundamental role in promoting migration of tumor cells (Zhu et al. 2016; Orozco et al. 2018), we then investigated whether MV-derived Gal-1 released from activated fibroblasts affects the migratory ability of DU145 and PANC-1 tumor cells.

It is noteworthy that MVs purified from normal fibroblasts are weakly able to increase DU145 migration respect to control, while MVs purified from activated fibroblasts strongly increase the migratory ability of tumor cells (Fig. 4). This effect correlates with enhanced Gal-1 expression in MVs derived from activated fibroblasts when compared to those derived from their normal counterpart (Fig. 1). This finding is consistent with our previous results, indicating that activated fibroblasts have a greater ability to transfer bioactive molecules via MVs-trafficking to recipient tumor cells, when compared to their not-activated counterpart (Santi et al. 2015).

Finally, we demonstrate that in a co-culture wound healing assay, Gal-1 stably silenced AHFs are far less efficient in promoting PANC-1 and DU145 cell migration than wild type AHFs, further substantiating the biological importance of exogenous Gal-1 in cancer cell migration (Fig. 5).

Accordingly, several studies highlight the key role of CAF-derived EVs in enhancing the migratory and invasive potential of recipient cancer cells. For example, Leca and co-authors revealed an increase in the migratory abilities of PDAC cells after the uptake of CAF-derived ANXA6 positive EVs (Leca et al. 2016). Similarly, EVs secreted by fibroblasts support colorectal cancer cell proliferation, probably through the transfer of amphiregulin (Oszvald et al. 2020), and FAK signaling in CAFs regulates the abilities of CAF-derived exosomes to induce breast cancer cell migration and ultimately metastasis (Wu et al. 2020). Coherently, Luga and co-workers reported that fibroblast-secreted exosomes drive breast cancer cell invasion through Wnt-planar cell polarity autocrine signaling (Luga et al. 2012). In addition, it has been demonstrated that CAF-derived EVs promote the migration and invasion of oral squamous cell carcinoma (Dourado et al. 2019). However, the importance of the specific transfer of proteins via MVs in affecting migration of recipient cells has not been largely addressed so far.

Overall, our work highlights the fundamental role of the MVs-mediated trafficking of specific CAF proteins in the crosstalk, within TME, between cancer and stromal cells, thereby providing specific phenotypic advantages to tumor cells. In our case, exogenous Gal-1, derived from MVs released by CAFs, increases cell motility in two different tumor cell lines. To the best of our knowledge this is the first example showing that an exogenous protein transferred from CAFs to cancer cells using MVs as vehicles may profoundly affect cancer progression.
